# Molecular and hematological characterization of thalassemia and hemoglobinopathies among pediatric patients in northern Lao People’s Democratic Republic

**DOI:** 10.1038/s41598-026-51509-8

**Published:** 2026-05-06

**Authors:** Volapheth Kanyasone, Pichpimon Pakpuak, Supitsaraporn Satewin, Anupong Pansuwan, Kritsada Singha, Attawut Chaibunruang, Hataichanok Srivorakun, Supan Fucharoen, Annkham Thammaseng, Sengmany Sengphachan, Hame Thiphalack, Anousine Phonedala, Supawadee Yamsri

**Affiliations:** 1 Medical Science Program, Graduate school, Khon Kaen University, Khon Kaen, Thailand; 2 Bachelor of Science Program in Medical Technology, Faculty of Associated Medical Sciences, Khon Kaen University, Khon Kaen, Thailand; 3 Centre for Research and Development of Medical Diagnostic Laboratories, Faculty of Associated Medical Sciences, Khon Kaen University, Khon Kaen, Thailand; 4 Faculty of Medicine, Mahasarakham University, Mahasarakham, Thailand; 5 Lao Friends Hospital for Children, Luang Prabang, Lao People’s Democratic Republic

**Keywords:** Thalassemia, Hemoglobinopathies, Pediatric, Anemia, Molecular diagnosis, Lao PDR, Diseases, Genetics, Medical research, Molecular biology, Molecular medicine

## Abstract

Thalassemia and hemoglobinopathies are highly prevalent in the Lao People’s Democratic Republic (Lao PDR). Luang Prabang Province represents an ethnically diverse area with a high burden of pediatric anemia, but the molecular and hematological characteristics of thalassemia in this setting have not been systematically described. This hospital-based cross-sectional study included consecutive pediatric patients encountered at the Lao Friends Hospital for Children, Luang Prabang, between August and November 2025. Leftover EDTA-anticoagulated blood specimens and corresponding clinical data were analyzed. Hemoglobin analysis was performed using capillary zone electrophoresis, and molecular characterization of α- and β-globin gene defects was conducted using PCR-based assays. A total of 206 pediatric patients were recruited. β-thalassemia disease accounted for 51.9% of hospital presentations (n = 107), while 26.2% of patients had α-thalassemia diseases (n = 54), and the remaining cases comprised combined α- and β-thalassemia diseases (n = 38, 18.9%). Molecular analysis identified as many as 40 distinct thalassemia genotypes, indicating substantial genetic heterogeneity. Among α-thalassemia disease, non-deletional Hb H disease, predominantly associated with the --^SEA^/α^CS^α genotype, was the most frequent presentation. Among β-thalassemia disease, β⁰/β^E^ was the most common genotype. At the molecular level, hemoglobin E was the most frequent β-globin allele, followed by the codons 41/42 (-TTCT), codon 17 (A > T), and promoter − 28 mutations (A > G). Hematological findings demonstrated moderate to severe anemia across clinically significant thalassemia syndromes. The first hospital-based molecular and hematological characterization of pediatric thalassemia in northern Lao PDR demonstrates genotypic and phenotypic heterogeneities, with clinically significant β-thalassemia and non-deletional α-thalassemia. These findings provide important evidence to support more accurate diagnostic approaches and inform thalassemia screening and prevention strategies in Lao PDR.

## Introduction

Thalassemia is a group of inherited autosomal recessive disorders caused by reduced or absent synthesis of globin chains, leading to chronic anemia with variable clinical severity. It is one of the most common monogenic diseases worldwide and is highly prevalent in tropical and subtropical regions, particularly in Southeast Asia^1,2^. Thalassemia is broadly classified into α- and β-thalassemia and is clinically categorized as transfusion-dependent thalassemia (TDT) and non–transfusion-dependent thalassemia (NTDT). Disease severity depends on the number and type of affected genes, with TDT requiring regular lifelong transfusions, whereas NTDT is associated with variable clinical severity and does not require regular transfusion^3^.

The Lao PDR is a landlocked country in Southeast Asia where thalassemia represents a significant public health concern. Most available epidemiological and molecular data have originated only from regions with relatively developed healthcare infrastructure, particularly the capital city, Vientiane. Studies from central Laos have reported an α⁰-thalassemia prevalence of approximately 12.0–14.0%. The reported carrier frequency of α⁺-thalassemia due to the 3.7 kb deletional α^+^-thalassemia ranges from 8.4% to 27.2%. Among non-deletional α⁺-thalassemia variants, hemoglobin Constant Spring (Hb CS) has been reported at frequencies of 4.5%−7.8%, whereas Hb Paksé is much less common, with a reported frequency of approximately 0.3%^4–7^. Interactions among these α-globin gene defects contribute to clinically significant diseases, including Hb H disease and AEBart’s disease, which are associated with microcytic hypochromic anemia and, in some cases, transfusion requirements^8^. The most severe form of α-thalassemia, homozygous α⁰-thalassemia, results in hemoglobin Bart’s hydrops fetalis syndrome^1^.

In β-thalassemia, disease severity varies according to the underlying β-globin gene mutations and their interaction with other Hb variants. Common β-globin gene mutations reported in Lao PDR include codon 41/42 (–TTCT), codon 17 (A > T), Hb E (G > A), and the promoter − 28 (A > G) mutation. The prevalence of β-thalassemia in central Laos has been estimated at approximately 2.3–4.3%, while Hb E is highly prevalent, affecting 20.0–30.2% of the population^5–7^. These variants contribute to severe disease phenotypes such as homozygous β⁰-thalassemia and β-thalassemia/Hb E. In addition, complex thalassemia intermedia phenotypes, including δβ⁰-thalassemia co-inherited with Hb E, have been described among certain ethnic minority groups^9^. These thalassemia syndromes constitute major clinical and public health challenges in Laos. However, comprehensive data on thalassemia prevalence and its molecular basis in other regions of Laos, particularly the northern areas, remain limited. This lack of information poses a significant challenge for developing effective prevention and control strategies across the country.

Luang Prabang Province, located approximately 350 km north of Vientiane, is characterized by marked ethnic diversity, including Lao Loum, Lao Thung, and Lao Soung populations, as well as ethnic minority groups such as Khmu, Hmong, and Akha^10^. Recent reports have documented a high prevalence of anemia in this region, affecting approximately 30.7% of women and 55% of pregnant women, indicating a substantial public health burden^11^. Inherited hemoglobin disorders are likely to contribute to anemia in this population; however, molecular evidence supporting this association is limited. Despite the high prevalence of anemia and the clinical burden of suspected thalassemia in Luang Prabang, no molecular characterization of thalassemia and hemoglobinopathies in this region has been reported to date, leaving the molecular basis of thalassemia in northern Laos, including Luang Prabang, largely unknown.

The Lao Friends Children’s Hospital (LFHC) in Luang Prabang serves as a major pediatric referral center for northern Laos and manages a large number of children with suspected thalassemia. At present, diagnosis at LFHC relies primarily on clinical assessment, complete blood count parameters, and hemoglobin analysis, with limited access to molecular testing. Consequently, definitive genotypic diagnoses for many patients remain unavailable. Characterizing the molecular basis of thalassemia and hemoglobinopathies among pediatric patients treated at LFHC is essential for improving diagnostic accuracy, guiding appropriate clinical management, enabling genetic counseling, and supporting the development of effective thalassemia prevention and control strategies in Lao PDR.

## Materials and methods

### Study design and population

This cross-sectional study was conducted among pediatric patients diagnosed with thalassemia and hemoglobinopathies who received clinical care at the thalassemia clinic of the Lao Friends Hospital for Children (LFHC), Luang Prabang, Lao PDR. All consecutive pediatric patients presenting to the thalassemia clinic, LFHC between August and November 2025 who met the eligibility criteria and provided informed consent were enrolled. The study utilized leftover EDTA-anticoagulated blood specimens and corresponding laboratory and clinical data collected through routine diagnostic services.

Although LFHC provides pediatric care for patients up to 18 years of age, in routine clinical practice, the thalassemia clinic predominantly manages patients younger than 15 years. Therefore, all patients included in this study were younger than 15 years. Patients were eligible if they had been diagnosed as having thalassemia or suspected hemoglobinopathies based on clinical assessment, complete blood count (CBC) results, red blood cell morphology, and hemoglobin analysis performed at LFHC. Patients were excluded if residual EDTA-anticoagulated blood specimens were unavailable or insufficient for further laboratory investigation.

The study was conducted in accordance with ethical guidelines for research involving human participants. Leftover clinical specimens obtained during routine patient care were used. Written informed consent for the use of these leftover specimens was obtained from patients and/or their legal guardians prior to study enrollment. The study protocol was reviewed and approved by the National Ethics Committee for Health Research (NECHR), Ministry of Health, Lao People’s Democratic Republic (Approval No. 50/NECHR; Submission ID 2025.15; approved on 17 January 2025), and by the Center for Ethics in Human Research, Khon Kaen University, Thailand (IRB No. IRB00008614; Approval No. HE682060; approved on 11 April 2025). Participant confidentiality was maintained through data anonymization prior to analysis.

### Specimens and data collection

Leftover EDTA-anticoagulated whole blood specimens obtained during routine clinical care were used for this study. After completion of routine diagnostic testing at LFHC, leftover specimens were retained for further investigation. Clinical and laboratory data, including age, hematological parameters, hemoglobin analysis results, final clinical diagnosis, and available transfusion history, were retrieved from the hospital database. All blood samples were collected at the time of hospital presentation under steady-state conditions. In routine clinical practice, blood samples for complete blood count (CBC) and related investigations are collected prior to any red blood cell transfusion to assess the severity of anemia and guide clinical management. Therefore, all hematological parameters analyzed in this study represent pre-transfusion values.

All leftover specimens were transported to the Thalassemia Service Unit (TSU), Centre for Research and Development of Medical Diagnostic Laboratories (CMDL), Faculty of Associated Medical Sciences, Khon Kaen University, Thailand, for confirmatory and molecular analyses of thalassemia.

### Thalassemia and hemoglobinopathies investigations

All participants underwent hemoglobin analysis and quantification using capillary zone electrophoresis (Capillarys system; Sebia, Lisses, France) according to the manufacturer’s instructions. Genomic DNA was extracted from peripheral EDTA-anticoagulated whole blood using the GT-Blood DNA Extraction Kit (RBC Bioscience, Taipei, Taiwan), following the manufacturer’s protocol.

A stepwise molecular diagnostic approach was applied. Based on hemoglobin analysis results, participants with profiles suggestive of thalassemia or hemoglobinopathies were further investigated using PCR-based methods.

For β-globin gene analysis, common β-thalassemia point mutations were screened using multiplex allele-specific polymerase chain reaction (ASPCR), including codons 41/42 (–TTCT) (*HBB*: c.126_129delCTTT), codon 17 (A > T) (*HBB*: c.52 A > T), IVS I-5 (G > T) (*HBB*: c.92 + 5G > T), IVS I-1 (G > T) (*HBB*: c.92 + 1G > T), IVS II-654 (C > T) (*HBB*: c.316–197 C > T), promoter − 28 (A > G) (*HBB*: c.−78 A > G), codons 71/72 (+ A) (*HBB*: c.216_217insA), codon 26 (G > T) (*HBB*: c.79G > T), and codon 26 (G > A) (*HBB*: c.79G > A; hemoglobin E). Common deletions, including the 3.4 kb deletion (NC_000011.10: g.5224302_5227791del), Filipino deletion (NC_000011.10: g.5112882_5231358del), and Prachinburi deletion (NC_000011.10: g.5167971_5228123delinsA), were identified using gap-PCR. In addition, 21 rare β-thalassemia mutations were analyzed using PCR-based assays routinely implemented in the TSU workflow^12,13^.

For α-globin gene analysis, all participants were screened for common α-thalassemia deletions. Specifically, α⁰-thalassemia deletions (--^SEA^) (NC_000016.10:g.165401-184701del) and (--^THAI^) (NC_000016.10:g.149863-183312del) and α⁺-thalassemia deletions (-α³·⁷) (NC_000016.10: g.149863_183312del) and (-α⁴·²) (NC_000016.10: g.169818_174075del) were identified using multiplex gap-PCR, whereas non-deletional variants, including Hb CS (*HBA2*: c.427T > C) and Hb PS (*HBA2*: c.429 A > T), were detected using ASPCR^14,15^. Participants with uncharacterized mutation were further analyzed by direct DNA sequencing using an ABI PRISM™ 3730 XL DNA Analyzer.

### Classification of thalassemia disease categories

Patients were classified based on thalassemia genotypes into two main groups: thalassemia diseases and non-thalassemia diseases. The thalassemia diseases group included individuals with genotypes associated with clinically significant thalassemia and was further categorized into β-thalassemia diseases, α-thalassemia diseases, and combined α- and β-thalassemia diseases according to the underlying genotypes. In contrast, the non-thalassemia diseases group comprised individuals without pathogenic α- or β-globin gene defects consistent with thalassemia. Although these individuals presented with clinical and hematological features suggestive of thalassemia, molecular analysis did not support a diagnosis of thalassemia.

Patients were further categorized as transfusion-dependent thalassemia (TDT) or non–transfusion-dependent thalassemia (NTDT) based on transfusion requirements and clinical severity, as determined by the treating physicians. Patients who required regular red blood cell transfusions for survival or maintenance of adequate hemoglobin levels were classified as TDT, whereas those who did not require regular transfusions or required only occasional transfusions were classified as NTDT, in accordance with standard clinical definitions.

### Statistical analysis

Statistical analyses were performed using SPSS version 26.0 (IBM Corp., Armonk, NY, USA). Continuous variables were summarized using descriptive statistics, and allele frequencies were calculated and presented as percentages with 95% confidence intervals (CI).

## Results

### Distribution of thalassemia disease categories

**Fig. 1:**
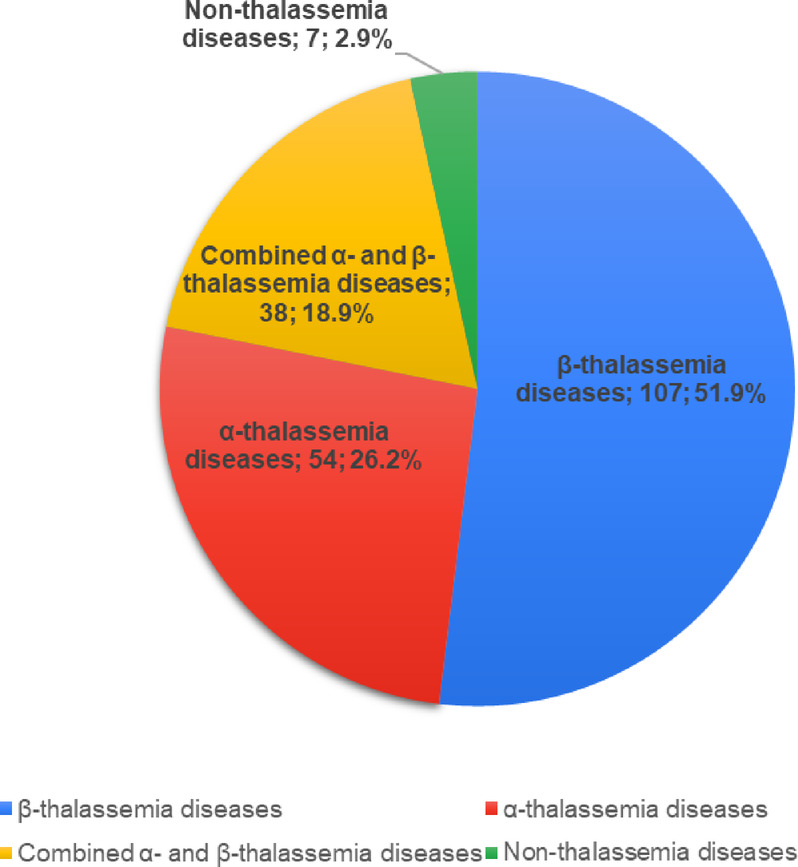
Distribution of thalassemia disease categories among pediatric patients (*n* = 206). The pie chart illustrates the proportions of β-thalassemia diseases (*n* = 107, 51.9%), α-thalassemia diseases (*n* = 54, 26.2%), combined α- and β-thalassemia diseases (*n* = 38, 18.9%), and non-thalassemia diseases (*n* = 7, 2.9%) identified in the study cohort.

In this hospital-based cohort, a total of 206 pediatric patients aged 0.7–15 years were recruited in the analysis. The median age of the patients was 8.0 years (IQR, 5.0–11.0). β-thalassemia diseases accounted for the largest proportion of cases (*n* = 107, 51.9%), followed by α-thalassemia diseases (*n* = 54, 26.2%). Combined α- and β-thalassemia diseases were identified in 38 patients (18.9%), while non-thalassemia diseases were observed in 7 patients (2.9%) (Fig. 1).

## Thalassemia genotypes among pediatric patients

In this study cohort, molecular analysis identified as many as 40 thalassemia genotypes. All participants were successfully characterized at the molecular level using the applied stepwise molecular diagnostic approach, and no cases remained uncharacterized after complete analysis. The detailed distribution of genotypes within each disease category is summarized in Table 1. Among patients with β-thalassemia diseases (*n* = 107), β^0^/β^E^ was the most frequently observed genotype, identified in 82 patients (76.6%). Within this group, the most common genotypes were αα/αα, β^17^/β^E^ (*n* = 38) and αα/αα, β^41/42^/β^E^ (*n* = 33). Other β⁰/βᴱ genotypes, including codons 71/72 (+ A), IVSI-5 (G-C), codon 27 (+ C), and codon 95 (+ A) mutations, were observed at lower frequencies. β⁰/β⁰ and β⁰/β⁺ genotypes accounted for 14 (13.1%) and 9 cases (8.4%), respectively, while β⁺/βᴱ genotypes were identified in 2 patients (1.9%).

**Table 1:** Thalassemia genotypes identified among pediatric patients (*n* = 206).

Thalassemia types	Genotypes	*n*
β-thalassemia diseases (*n* = 107)		
β^0^/β^E^ (*n* = 82; 76.6%)	αα/αα, β^17^/β^E^	38
	αα/αα, β^41/42^/β^E^	33
	αα/αα, β^71/72^/β^E^	8
	αα/αα, β^IVSI−5^/β^E^	1
	αα/αα, β ^27^/β^E^	1
	αα/αα, β^95^/β^E^	1
β^+^/β^E^ (*n* = 2; 1.9%)	αα/αα, β^−28^/β^E^	2
β^0^/β^0^ (*n* = 14; 13.1%)	αα/αα, β^41/42^/β^17^	6
	αα/αα, β^41/42^/β^41/42^	3
	αα/αα, β^17^/β^17^	3
	αα/αα, β^41/42^/β^71/72^	1
	αα/αα, β^71/72^/β^71/72^	1
β^0^/β^+^ (*n* = 9; 8.4%)	αα/αα, β^41/42^/β^−28^	7
	αα/αα, β^17^/β^−28^	2
α-thalassemia diseases (*n* = 54)		
Non deletion-Hb H (*n* = 51; 94.4%)	--^SEA^/α^CS^α, β^A^/β^A^	48
	--^THAI^/α^CS^α, β^A^/β^A^	1
	--^SEA^/α^QS^α, β^A^/β^A^	1
	--^SEA^/α ^Init CD (−T)^α, β^A^/β^A^	1
Hb H (*n* = 3; 5.6%)	--^SEA^/-α^3.7^, β^A^/β^A^	3
α- and β-thalassemia diseases (*n* = 38)		
β^0^/β^E^ (*n* = 8; 21.1%)	αα/-α^3.7^, β^17^/β^E^	3
	αα/-α^3.7^, β^41/42^/β^E^	2
	αα/-α^4.2^, β^41/42^/β^E^	1
	αα/--^SEA^, β^41/42^/β^E^	2
β^0^/β^+^ (*n* = 3; 7.9%)	--^SEA^/-α^3.7^, β^41/42^/β^−28^	1
	αα/α^CS^α, β^17^/β^−28^	1
	αα/-α^3.7^, β^41/42^/β^−28^	1
β^0^/β^0^ (*n* = 8; 21.1%)	αα/-α^3.7^, β^41/42^/β^17^	1
	αα/-α^4.2^, β^41/42^/β^17^	1
	αα/α^CS^α, β^41/42^/β^17^	1
	--^SEA^/αα, β^41/42^/β^17^	1
	αα/-α^3.7^, β^41/42^/β^41/42^	1
	αα/--^SEA^,β^41/42^/β^41/42^	1
	αα/-α^3.7^, β^17^/β^17^	1
	αα/--^SEA^, β^17^/β^17^	1
AEBart’s-CS (*n* = 18; 47.4%)	--^SEA^/α^CS^α, β^E^/β^A^	18
Heterozygous Hb E with homozygous Hb CS (*n* = 1; 2.6%)	α^CS^α/α^CS^α, β^E^/β^A^	1
Non-thalassemia diseases (*n* = 7)		
Homozygous Hb E (*n* = 4; 57.1%)	αα/αα, β^E^/β^E^	3
	αα/--^SEA^, β^E^/β^E^	1
Heterozygous β-thal (*n* = 1; 14.3%)	αα/--^SEA^, β^41/42^/β^A^	1
Heterozygous Hb CS (*n* = 1; 14.3%)	αα/α^CS^α, β^A^/β^A^	1
Non-thalassemia (*n* = 1; 14.3%)	αα/αα, β^A^/β^A^	1

Among α-thalassemia diseases (*n* = 54), non-deletional Hb H disease was the most frequently observed genotype group, identified in 51 patients (94.4%), predominantly associated with the --^SEA^/α^CS^α genotype. Deletional Hb H disease (--^SEA^/-α^3.7^) was identified in 3 patients (5.6%).

Patients with combined α- and β-thalassemia diseases (*n* = 38) demonstrated marked genotypic heterogeneity. β⁰/βᴱ and β⁰/β⁰ genotypes were each identified in 8 patients (21.1%), while β⁰/β⁺ genotypes were observed in 3 patients (7.9%). The AEBart’s disease (Hb H disease with Hb E trait) associated with Hb CS (--^SEA^/α^CS^α, β^E^/β^A^) was identified in 18 patients (47.4%).

Among non-thalassemia diseases group (*n* = 7), homozygous Hb E was the most frequent genotype (*n* = 4), followed by each single case of heterozygous β-thalassemia, and heterozygous Hb CS, and a normal globin genotype.

### Spectrum and allele frequencies of globin gene defects

The spectrum and relative frequencies of α- and β-globin gene defects identified among the study cohort are summarized in Table 2. A total of 412 alleles were analyzed based on molecular characterization. Among α-globin gene defects, the --^SEA^ deletion was the most frequent allele, accounting for 19.2% of identified alleles (95% CI: 15.7–23.3%), followed by Hb CS (17.5%, 95% CI: 14.1–21.4%). Other α-globin defects, including the -α³·⁷ deletion (3.2%, 95% CI: 1.9–5.3%) and -α⁴·² deletion (0.5%, 95% CI: 0.06–1.74%), were observed at lower frequencies. Rare non-deletional α-globin variants, including Hb Quong Sze (*HBA2*: c.377T > C) and the α-^Init CD (−T)^ (*HBA2*: c.2delT), were each identified at a frequency of 0.2% (95% CI: 0.01–1.34%) and were confirmed by Sanger sequencing.

**Table 2:** Spectrum and relative frequencies of α- and β-globin gene defects identified among pediatric patients.

Gene	Gene defect	Alleles*, *n*	%	95% CI
α-globin	--^SEA^ deletion	79	19.2	15.7–23.3
	Hb Constant Spring	72	17.5	14.1–21.4
	-α³·⁷ deletion	13	3.2	1.9–5.3
	-α⁴·² deletion	2	0.5	0.06–1.74
	--^THAI^ deletion	1	0.2	0.01–1.34
	Hb Quong Sze	1	0.2	0.01–1.34
	α-^Init CD (−T)^	1	0.2	0.01–1.34
β-globin	Hb E (Codon 26, G > A)	119	28.9	24.6–33.5
	Codon 41/42 (–TTCT)	69	16.7	13.3–20.7
	Codon 17 (A > T)	64	15.5	12.2–19.4
	–28 (A > G)	14	3.4	1.9–5.6
	Codon 71/72 (+ A)	11	2.7	1.3–4.7
	IVSI-5 (G > C)	1	0.2	0.01–1.34
	Codon 27 (+ C)	1	0.2	0.01–1.34
	Codon 95 (+ A)	1	0.2	0.01–1.34

*Allele frequencies were calculated from all alleles identified (*n* = 412 alleles) in Table 1. Normal alleles not shown in the table accounted for 243 α-globin alleles (59.0%) and 132 β-globin alleles (32.0%).

Among β-globin gene defects, Hb E (codon 26, G > A) was as expected the most frequently observed allele, accounting for 28.9% of identified alleles (95% CI: 24.6–33.5%). This was followed by the codons 41/42 (–TTCT) mutation (16.7%, 95% CI: 13.3–20.7%) and the codon 17 (A > T) mutation (15.5%, 95% CI: 12.2–19.4%). Other β-thalassemia alleles, including the − 28 (A > G) promoter mutation and codon 71/72 (+ A), were detected at lower frequencies (3.4%, 95% CI:1.9–5.6% and 2.7%, 95% CI:1.3–4.7, respectively) while rare β-globin mutations were each identified in a small number of alleles (Table 2).

### Hematological parameters and anemia severity in Hb H and AEBart’s diseases

Patients with Hb H-CS had a median Hb level of 8.0 (IQR, 7.0–8.7) g/dL, a mean corpuscular volume (MCV) of 71.0 (IQR, 67.4–74.0) fL, and a mean corpuscular hemoglobin (MCH) of 19.0 (IQR, 18.0–20.0) pg. Based on Hb concentration, most patients in this group were classified as having moderate to severe anemia. Patients with AEBart’s disease associated with Hb CS (CS-AEBart’s) showed a median Hb level of 7.7 (IQR, 7.0–8.0) g/dL, MCV of 68.5 (IQR, 58.0–73.0) fL, and MCH of 18.3 (IQR, 16.9–19.0) pg, consistent with predominantly moderate anemia and microcytic, hypochromic red blood cell indices. Patients with deletional Hb H disease (--^SEA^/-α³·⁷) demonstrated hematological parameters comparable to those observed in non-deletional Hb H disease, with a median Hb level of 8.0 (IQR, 7.5–8.5) g/dL, MCV of 59.0 (IQR, 56.0–60.0) fL, and MCH of 16.0 (IQR, 15.5–16.5) pg. Rare Hb H genotypes, including Hb H-QS and Hb H-α-^Init CD (−T)^, were each observed as single case and demonstrated moderate anemia with microcytic and hypochromic indices (Table 3).

**Table 3:** Hematological parameters among patients with Hb H and AEBart’s diseases with different genotypes.

Thalassemia disease	*N* (%)	Age (year)	Rbc (x10^12^/L)	Hb (g/dL)	HCT (%)	MCV (fL)	MCH (pg)	RDW-CV (%)
Hb H-CS	49	8.0 (4.0–10.0)	4.2 (3.8–4.7)	8.0 (7.0–8.7)	29.0 (27.3–32.0)	71.0 (67.4–74.0)	19.0 (18.0–20.0)	22.9 (22.0–27.0)
Hb H-QS	1	12	6.4	10.0	37.0	58.0	16.0	23.0
Hb H-Init CD ^(−T)^	1	8	3.8	6.9	27.9	73.0	18.1	34.0
Hb H	3	8.0 (5.0–8.5)	5.4 (4.7–5.4)	8.0 (7.5–8.5)	29.0 (27.0–30.5)	59.0 (56.0–60.0)	16.0 (15.5–16.5)	24.0 (22.5–27.3)
AEBart’s-CS	18	10.0 (5.3–11.8)	4.3 (4.1–4.4)	7.7 (7.0–8.0)	27.7 (27.0–28.8)	68.5 (58.0–73.0)	18.3 (16.9–19.0)	23.9 (22.4–25.1)

Values are presented as median (IQR) or as raw data where appropriate.

### Hematological parameters and anemia severity in β-thalassemia patients

Hematological parameters of pediatric patients with β-thalassemia stratified by β-globin genotype and α-globin gene status are summarized in Table 4. Among patients with β⁰/β^E^ and a normal α-globin genotype (*n* = 82), the median Hb level was 6.8 (IQR, 6.0–7.4) g/dL, with a MCV of 66.0 (IQR, 63.0–70.8) fL and a MCH of 20.6 (IQR, 19.1–22.0) pg, consistent with moderate to severe anemia. Patients with β⁰/β^E^ carrying one α-globin gene defect (*n* = 6) showed a median Hb level of 7.1 (IQR, 6.9–7.8) g/dL, MCV of 56.1 (IQR, 52.6–59.8) fL, and MCH of 16.2 (IQR, 15.1–17.9) pg. Two patients with β⁰/β^E^ with two α-globin gene defects exhibited Hb levels of 7.0 and 5.0 g/dL, with corresponding MCVs of 63.2 and 60.0 fL and MCH values of 18.8 and 19.0 pg, respectively.

**Table 4:** Hematological parameters of pediatric patients with β-thalassemia according to α-globin gene status.

β-thalassemia genotypes	α-globin genes	*N* (%)	Age (year)	Rbc (x10^12^/L)	Hb (g/dL)	HCT (%)	MCV (fL)	MCH (pg)	RDW-CV (%)
β^0^/β^E^	Normal	82	8.0 (6.0–11.0)	3.2 (3.0–3.7)	6.8 (6.0–7.4)	21.7 (19.3–24.2)	66.0 (63.0–70.8)	20.6 (19.1–22.0)	26.0 (23.0–28.8)
	1 α-gene defect	6	8.5 (2.5–10.8)	4.5 (4.4–4.7)	7.1 (6.9–7.8)	24.4 (23.3–25.7)	56.1 (52.6–59.8)	16.2 (15.1–17.9)	27.8 (23.3–29.7)
	2 α-gene defects	2	9.0, 6.0	3.7, 2.9	7.0, 5.0	23.2,17.0	63.2, 60.0	18.8, 19.0	26.6, 35.9
β^0^/β^0^	Normal	14	5.5 (3.0–10.0)	2.4 (2.3–2.8)	6.0 (5.5–6.9)	18.0 (16.6–21.8)	74.2 (71.0–76.0)	24.0 (22.8–25.0)	17.5 (15.2–21.0)
	1 α-gene defect	5	8.0 (7.0–10.0)	2.7 (2.5–2.8)	6.2 (5.8–6.8)	19.1 (18.1–21.0)	72.9 (72.1–74.0)	23.4 (23.1–24.0)	16.5 (15.1–17.3)
	2 α-gene defect	3	4.0 (3.5–7.5)	2.3 (2.2–2.6)	4.5 (4.5–6.1)	14.3 (14.2–18.7)	68.3 (65.5–73.1)	22.0 (20.7–23.8)	25.5 (19.9–26.7)
β^0^/β^+^	Normal	9	7.0 (5.0–8.0)	3.1 (2.9–3.2)	6.7 (6.0–7.0)	21.0 (20.0–22.1)	69.0 (67.0–72.0)	22.0 (21.0–23.4)	22.1 (20.0–24.6)
	1 α-gene defect	2	12.0, 12.0	2.0, 2.2	5.0, 4.0	14.6, 13.0	72.3, 61.0	22.8, 18.0	26.5, 36.5
	3 α-gene defect	1	5.0	3.5	9.0	28.0	78.0	26.0	16.0
β^+^/β^E^	Normal	2	10, 10	3.3, 5.1	8.0, 8.9	26.0, 26.6	77.0, 52.5	23.0, 17.6	23, 25.6

Values are presented as median (IQR) or as raw data where appropriate.

For patients with β⁰/β⁰, those with a normal α-globin genotype (*n* = 14) had a median Hb level of 6.0 (IQR, 5.5–6.9) g/dL, MCV of 74.2 (IQR, 71.0–76.0) fL, and MCH of 24.0 (IQR, 22.8–25.0) pg, while patients with one α-globin gene defect (*n* = 5) demonstrated comparable hematological profiles (Hb 6.2 (IQR, 5.8–6.8) g/dL, MCV 72.9 (IQR, 72.1–74.0) fL, and MCH 23.4 (IQR, 23.1–24.0) pg. Three patients with β⁰/β⁰ and two α-globin gene defects had a median Hb level of 4.5 (IQR, 4.5–6.1) g/dL, a MCV of 68.3 (IQR, 65.5–73.1) fL, and a MCH of 22.0 (IQR, 20.7–23.8) pg, consistent with moderate to severe anemia. Among patients with β⁰/β⁺ and a normal α-globin genotype (*n* = 9), the median Hb level was 6.7 (IQR, 6.0–7.0) g/dL, with a MCV of 69.0 (IQR, 67.0–72.0) fL and a MCH of 22.0 (IQR, 21.0–23.4) pg. Two patients with β⁰/β⁺ and one α-globin gene defect exhibited Hb levels of 5.0 and 4.0 g/dL, whereas a single patient with three α-globin gene defects had a Hb level of 9.0 g/dL. Patients with β⁺/βᴱ (*n* = 2) demonstrated moderate anemia, with Hb levels of 8.0 and 8.9 g/dL.

### Hematological characteristics of patients with non-thalassemia diseases

A total of seven patients were classified as having non-thalassemia diseases based on the absence of pathogenic α- or β-globin gene defects consistent with thalassemia (Table 5). Despite this, these patients presented with clinical and hematological features suggestive of thalassemia, including anemia and microcytic hypochromic red blood cell indices. Hemoglobin levels ranged from 6.0 to 11.0 g/dL, indicating mild to moderate anemia in most cases, with one patient presenting with more severe anemia. Red blood cell indices were consistent with microcytosis and hypochromia, with MCV ranging from 50.0 to 73.0 fL and MCH from 17.0 to 25.0 pg. RDW-CV values were variably elevated (11.0–24.3%), reflecting heterogeneity in red cell morphology.

**Table 5:** Hematological parameters of pediatric patients with non-thalassemia diseases.

Case no.	Thalassemia genotype	Age (year)	Rbc (x10^12^/L)	Hb (g/dL)	HCT (%)	MCV (fL)	MCH (pg)	RDW-CV (%)
1	αα/αα, β^E^/β^E^	8	5.1	10.0	31.0	62.0	20.0	16.6
2	αα/αα, β^E^/β^E^	8	5.7	10.6	34.3	60.5	18.7	14.7
3	αα/αα, β^E^/β^E^	3	5.7	10.0	29.0	50.0	17.0	19.2
4	αα/--^SEA^, β^E^/β^E^	4	5.9	10.0	34.0	57.0	17.0	16.6
5	αα/--^SEA^, β^41/42^/β^A^	1	6.2	11.0	33.0	54.0	17.0	24.3
6	αα/α^CS^α, β^A^/β^A^	7	4.8	11.0	34.0	70.0	22.0	11.0
7	αα/αα, β^A^/β^A^	3	2.4	6.0	18.0	73.0	25.0	17.0

## Discussion

This is the first hospital-based molecular and hematological study of thalassemia and hemoglobinopathies among pediatric patients in Luang Prabang Province, northern Lao PDR. The study provides insight into the clinically relevant spectrum of thalassemia diseases encountered. The distribution of disease categories observed in this cohort, as illustrated in Fig. 1, conveys an important clinical message. Approximately half of the pediatric patients presenting to the hospital were diagnosed with β-thalassemia diseases, while about 25% had α-thalassemia diseases, and the remaining patients exhibited combined α- and β-thalassemia diseases. This pattern reflects the relative contribution of different thalassemia syndromes to hospital-based disease burden in northern Laos. The predominance of β-thalassemia diseases among hospitalized children is consistent with the greater clinical severity and earlier presentation associated with β-globin gene defects, particularly β⁰ mutations and their interaction with Hb E. In contrast, α-thalassemia diseases are more likely to present to hospital in the presence of non-deletional variants, which result in clinically significant anemia^1^. As this was a hospital-based study conducted at a pediatric referral center, the observed distribution likely reflects clinically significant cases presenting for evaluation rather than population-level prevalence.

Within this hospital-based cohort, as many as 40 distinct thalassemia genotypes were identified among pediatric patients presenting to the hospital (Table 1), demonstrating that children presenting to the hospital in northern Lao PDR represent a highly heterogeneous spectrum of thalassemia syndromes. Both α- and β-thalassemia diseases were observed, together with a substantial proportion of complex αβ-thalassemia syndromes. This level of heterogeneity highlights the diagnostic challenges encountered in routine clinical settings.

Analysis of the globin gene defect spectrum (Table 2) indicates that the molecular basis of thalassemia among pediatric patients in northern Lao PDR is characterized by mutations commonly reported in Southeast Asia. Among α-globin gene defects, the --^SEA^ deletion was the most frequent allele, accounting for approximately 19.2% of α-globin abnormalities, followed by Hb CS, which represented about 17.5% of α-globin alleles. Population-based studies conducted in different regions of Lao PDR, particularly among pregnant women and specific ethnic groups in Vientiane Capital and surrounding provinces, have reported a high carrier frequency of α-thalassemia, ranging from approximately 9% to more than 30%. At the molecular level, these studies consistently identified the --^SEA^ deletion as the predominant α⁰-thalassemia allele and Hb CS as the most frequent non-deletional α-thalassemia variant. Similar mutation patterns have also been reported in neighboring Southeast Asian countries, including Thailand, Cambodia, and Vietnam, where the --SEA deletion is the predominant α⁰-thalassemia allele and Hb CS is one of the most common non-deletional variants. This regional consistency suggests a shared genetic background of α-globin mutations across Southeast Asia. However, while population surveys predominantly identify asymptomatic carriers, the present hospital-based pediatric cohort is enriched for clinically significant α-thalassemia, particularly non-deletional Hb H disease associated with Hb CS, which is more likely to result in symptomatic anemia and hospital presentation^2,4,6–8^.

Consistent with this molecular profile (Table 3), the hematological findings indicate that most hospitalized α-thalassemia patients had non-deletional Hb H disease, predominantly the --^SEA^/α^CS^α genotype. This genotype is more frequently associated with moderate to severe anemia and therefore leads to hospital presentation. In contrast, deletional Hb H disease, typically caused by the --^SEA^/-α^³·⁷^ genotype, was less frequently observed in this cohort and was generally associated with milder hematological abnormalities, supporting the observation that such patients are less likely to require hospital-based evaluation. This difference in clinical severity was also reflected in transfusion dependency patterns (data not shown). Most patients with deletional Hb H disease were classified as NTDT, whereas patients with Hb H-CS disease were predominantly classified as TDT. In AEBart’s disease associated with Hb CS, both NTDT and TDT phenotypes were observed, with NTDT predominating. The predominance of non-deletional Hb H disease among hospitalized patients is consistent with previous molecular studies in Lao PDR and northeast Thailand, which demonstrated a high contribution of Hb CS to clinically significant α-thalassemia syndromes^8,16^.

Moreover, rare non-deletional α-globin variants, including Hb QS and the α-^Init CD (−T)^, were identified in this cohort, both observed in patients with Hb H disease. Notably, the α-^Init CD (−T)^ was identified in a patient who presented with moderate anemia (Hb 6.9 g/dL) and microcytic hypochromic red cell indices, and was classified as non–transfusion-dependent thalassemia (NTDT). Collectively, these findings further expand the molecular spectrum of α-thalassemia in northern Lao PDR and underscore the importance of comprehensive molecular testing in this setting.

Among β-globin gene defects (Table 2), Hb E was the most prevalent allele, accounting for approximately 28.9% of β-globin mutations, followed by the codons 41/42 and codon 17 mutations, which together comprised a substantial proportion of the remaining β-thalassemia alleles. Consistent with population-based genetic studies in Lao PDR, which have reported a relatively low prevalence of β-thalassemia carriers (approximately 2–4%) but a high frequency of hemoglobin E (18–30%), hemoglobin E was also the most frequent β-globin allele identified in the present study. Similar mutation patterns have been widely reported in neighboring Southeast Asian countries, including Thailand, Cambodia, and Vietnam, where Hb E is highly prevalent and β⁰-thalassemia mutations such as codons 41/42 and codon 17 are among the most common disease-causing alleles. This regional consistency reflects a shared β-globin mutational background across the Greater Mekong Subregion^4,7,13,17^. In addition to β⁰-thalassemia mutations, the promoter − 28 (A > G) mutation was identified in this cohort and represents a common β⁺-thalassemia allele in northern Lao PDR; previous molecular studies in Lao populations and large cohort studies from Thailand have similarly reported this variant as one of the most frequent β⁺-thalassemia mutations, associated with variable hematological phenotypes and important diagnostic considerations^17,18^. Taken together, these findings support both the regional similarity in mutation patterns and the predominance of clinically significant β-thalassemia phenotypes in this hospital-based pediatric cohort.

As shown in Table 4, the hematological profiles demonstrated the variability among patients with β-thalassemia, depending on both the underlying β-globin genotype and the presence of co-inherited α-globin gene defects. While co-inheritance of α-thalassemia may partially modify globin chain imbalance in some patients, clinically significant anemia persisted across many genotype groups, underscoring the complexity of genotype–phenotype relationships in β-thalassemia^19,20^. In addition, the interpretation of hematological parameters in this hospital-based cohort should consider the impact of red blood cell transfusions, as a proportion of patients were receiving regular or intermittent transfusion therapy. Transfusions may transiently increase Hb levels and influence red cell indices, potentially masking the underlying severity of ineffective erythropoiesis and hemolysis^21^. Consequently, hematological measurements obtained in transfused patients may not fully reflect baseline disease severity, further contributing to the observed variability across genotype groups. Clinically, these genotype–phenotype differences were accompanied by differences in transfusion dependency. Patients with β⁰/β^E^ and β⁰/β⁰ genotypes accounted for the majority of TDT cases. Although co-inheritance of α-thalassemia was observed in some patients, TDT remained common in β-thalassemia disease, reflecting the greater clinical severity associated with β-globin gene defects (data not shown). Taken together, these findings indicate that hematological severity in β-thalassemia cannot be inferred from the β-globin genotype alone and suggest that additional genetic modifiers, transfusion history, and other clinical factors should be considered through integrated molecular and hematological assessment in clinical practice.

The presence of patients without clinically significant thalassemia syndromes in this hospital-based cohort, including individuals with homozygous Hb E, heterozygous β-thalassemia, heterozygous Hb CS, and those without identifiable globin gene defects (Table 5). A recent hospital-based study among women and young children in northern Lao PDR which reported a high prevalence of anemia associated with micronutrient deficiencies, including iron, thiamine, riboflavin, and vitamin A, as well as socioeconomic and health-related factors support this notion^11^. It is conceivable therefore that that children presenting to the hospital with anemia may present in association with nutritional deficiencies or acute illness, even in the absence of pathogenic globin gene mutations.

The mutation patterns identified in this study have important implications for disease control and prevention in Lao PDR. Their similarity to patterns reported in neighboring countries supports the feasibility of adapting screening strategies successfully implemented in Thailand. These include stepwise community-based screening using osmotic fragility testing or red cell indices (MCV/MCH), combined with the dichlorophenolindophenol (DCIP) testing for Hb E, followed by confirmatory hemoglobin analysis and targeted molecular testing for common mutations^22–24^. By documenting region-specific genetic patterns, this hospital-based study contributes essential data that can inform the development of national guidelines, resource allocation, and strategies for thalassemia screening and prevention in Lao PDR.

Nevertheless, several limitations should be considered. First, as a hospital-based study conducted at a pediatric referral center, the cohort reflects patients presenting for clinical evaluation rather than the general pediatric population in northern Lao PDR. Therefore, selection bias toward more clinically severe thalassemia phenotypes is likely, and milder forms and asymptomatic carriers may be under-represented. Second, although a comprehensive molecular diagnostic approach was applied, including targeted PCR assays for specific mutations and confirmatory sequencing for uncharacterized cases, the analysis was limited to α- and β-globin gene defects. Other genetic or environmental factors contributing to anemia, such as modifier genes or nutritional deficiencies, were not systematically evaluated. Finally, as the study was conducted at a single center, the findings may not be fully representative of all regions or ethnic groups in Lao PDR.

In conclusion, this first hospital-based molecular and hematological study from northern Lao PDR demonstrates that pediatric patients presenting to the hospital with anemia represent a clinically and genetically heterogeneous spectrum of thalassemia and hemoglobinopathies. The combined analysis of disease distribution, genotype diversity, allele frequencies, and hematological profiles shows that clinically significant β-thalassemia and non-deletional α-thalassemia account for a large proportion of hospital presentations. These results emphasize the importance of comprehensive molecular characterization in referral settings and provide a useful evidence base to support improved diagnostic approaches, screening strategies, and national planning for thalassemia control and prevention in Lao PDR.

## Data Availability

The datasets generated during the current study are not publicly available due to ethical and privacy restrictions. De-identified data are available from the corresponding author upon reasonable request and with institutional ethics approval. The Sanger sequencing data for rare α-globin variants identified in this study (Hb Quong Sze and α-initiation codon mutation) have been deposited in GenBank under accession numbers PZ324723-PZ324724.
